# Supplementary material for: In Search of a Target Gene for a Desirable Phenotype in Aquaculture: Genome Editing of Cyprinidae and Salmonidae Species
Source: Genes (Basel). 2024 Jun 1;15(6):726. doi: 10.3390/genes15060726 (PMC11202958; doi:10.3390/genes15060726)
Supplement: Supplementary file 1 [file genes-15-00726-s001.zip › genes-3004487-supplementary.pdf]

In search of target gene for desirable phenotype in aquaculture: genome editing of Cyprinidae and Salmonidae species.

Svetlana Yu. Orlova, Maria N. Ruzina, Olga R. Emelianova, Alexey A. Sergeev, Evgeniya A. Chkurova, Alexei M. Orlov, and Nikolai S. Mugue

Supplementary materials

Table S1. Summary of fish genome editing experiments affecting genes controlling economically valuable traits

Species	Gene	Protein	Method	Purpose of modification	New phenotype	Identified gene function	Source
**Zebrafish**	*hoxb5b*	Homeobox gene	CRISPR/Cas9	Development	Disruption in embryogenesis of the morphogenesis of the foregut endoderm, the location of tissue along the midline of the body, the formation of a bifurcated foregut, the absence of a liver	Launching the mechanism of foregut morphogenesis, which determines the location of the central line of the body	[221]
	foxc1a<br>*foxc1b*	Forkhead box C1a	CRISPR/Cas9	Development	Disruption of the formation of the cardiac loop in embryogenesis, abnormal position of the heart, isomerism (mirror arrangement) of the liver and pancreas, which entails incorrect position of the intestines, disturbances in the location of the visceral organs	Formation of symmetry relative to the central axis of the body	[222]
	bmp7a	Bone morphogenetic protein 7	CRISPR/Cas9	Development	In *bmp7a* mutants, incorrect formation of the dorsoventral axis was observed during embryogenesis, which led to their death	The bmp7a gene controls the formation of the dorsoventral axis.	[223]

gne	Glucosamine (UDP-N-acetyl)-2-epimerase	CRISPR/Cas9	Development	Death of larvae on the 8-10th day after fertilization, curvature of the body axis, reduction in the size of the swim bladder, decreased motor activity, decreased heart rate, disruption of the organization of muscle fibers, disruption of the cell cycle, and DNA repair processes	Control of the formation of body symmetry and muscle fibers	[224]
LOC795232	Protein from the natterine-like gene family	CRISPR/Cas9	Development	Delayed epiboly (migration of embryonic cells), swelling of the yolk sac and heart, swim bladder defects, reduction in the size of the eyes and head, behavioral disorders	Control of organogenesis, including the swim bladder	[225]
sphk1	Sphingosine kinase-1	CRISPR/Cas9	Development	Disorders of embryonic development - darkening and atrophy of the head, deformation of the body, pericardial edema, delayed development of the yolk sac, decreased heart rate, increased overall mortality of fry, increased expression of acetylcholinesterase, impaired expression of genes that control the development of the nervous system and the synthesis of neurotransmitters, impaired gene expression, associated with immunity, which led to a significant decrease in the number of hematopoietic stem cells and neutrophils, a decrease in the expression of genes responsible for heart development	Control of embryogenesis, including body shape, organogenesis, immunity	[226]

	asap1a<br>*asap1b*	ArfGAP with SH3 domain, ankyrin repeat and PH domain 1a	CRISPR/Cas9	Development	Reduced survival and hatching of fry, developmental defects in the early stages, impaired migration of neutrophils to the bacteria *Mycobacterium marinum*, increased bacterial load	Control of embryogenesis and immune response	[227]
	zrsr2	CCC-type zinc finger, RNA binding motif 2, rich in serine and arginine	CRISPR/Cas9	Development	On the fourth day after fertilization, multiple developmental defects appeared, and on the eighth day, all the embryos died. Transcriptomic analysis showed repression of genes of the most important metabolic pathways and retention of minor introns of some gene.	Monitoring the vitality of the organism	[228]
	slincR for sox9	Long intergenic non-coding RNA for SRY-related transcription factor 9	CRISPR/Cas9	Development	In mutant fish, an 18-bp insertion was made into the region of slincR, a long intergenic non-coding RNA for the *sox9* gene, which led to a change in its secondary structure. The resulting fish were cultured in the presence or absence of ligands for *sox9*, the aryl hydrocarbon receptor of AHR. The mutants were sensitive to the amount of AHR in the environment. In the absence of AHR, animals exhibited disruption of signaling pathways. In both cases, there were	Formation of cartilage tissue	[229]

				disturbances in the development of cartilage tissue and a decrease in the regenerative activity of the caudal fin, associated with the suppression of cell proliferation		
vwa1	von Willebrand factor domain A1	CRISPR/Cas9	Development	Defects of cartilage development, including Meckel's cartilage and palatoquadrate cartilage, widened angle deformation of ceratochial cartilage, deformation or absence of ceratobranchial cartilages, reduced chondrocyte size, change in shape and location. Impaired proliferation and survival of neural crest cells, decreased expression of the *barx1* and *col2a1a* genes, which indicates a violation of the condensation and differentiation of these cells. Reduced expression of the *fgf8a, fgfr1, fgfr2, fgfr3, fgfr4, runx2a* genes, which modulate signal transmission along the FGF (fibroblast growth factor) pathway	Control of cartilage tissue development, modulation of FGF signaling	[230]
tmem38b	Transmembrane protein 38B	CRISPR/Cas9	Development	Collagen 1 is not sufficiently formed, remains in the ER, decreases the length of the vertebrae, decreases the activity of osteoblasts and osteoclasts, disrupts the mineralization processes during regeneration of the caudal fin	Control of the formation of the skeleton and bone tissue	[231]

ppp2r3b	Protein phosphatase 2, regulatory subunit B, β isoform	CRISPR/Cas9	Development	Progressive kyphoscoliosis, decreased mineralization of the vertebrae, osteoporosis, mitochondrial defects in muscle fibers	Control of skeletal formation, bone mineralization	[68]
col11a2	Collagen, type XI, α2	CRISPR/Cas9	Development	Reduction of vertebrae in the caudal spine, mineralization of intervertebral segments	Formation of the spine, mineralization of cartilage	[69]
wnt16	Wingless-type MMTV integration site family, member 16	CRISPR/Cas9	Development	Decreased bone mineral density, spontaneous fractures, difficult recruitment and differentiation of osteoblasts to restore bones after fractures, accumulation of calluses, damage to lepidotrichia (bony fin rays)	Bone restoration after fractures by modulating osteoblast differentiation	[232]
wnt16	Wingless-type MMTV integration site family, member 16	CRISPR/Cas9	Development	Deformation of the skull, spine, tail, decreased bone mineral density, decreased expression of the genes *akt1, bnip4, ptena, vegfaa, twsg1b, prkab1a, prkab1b, pla2g4f.2*	Control of skeletal formation	[233]
hoxaa<br>*hoxab*<br>*hoxba*<br>*hoxba*<br>*hoxbb*<br>*hoxca*<br>*hoxcb*	Homeobox genes	CRISPR/Cas9	Development	The most serious developmental disorders were observed in mutants of the *hoxba* gene: defects in the formation of the brain, jaw, pectoral fins, and lateral line. The number of somites was increased in *hoxca* and *hoxab* mutants. *hoxca* mutants exhibited spinal deformity and abnormalities of the Weberian apparatus and swim bladder. *hoxda* mutants exhibited slight vertebral deformity. The *hoxaa* and *hoxab* mutants had	Control of the formation of the musculoskeletal system in embryogenesis	[234]

				shortened pectoral fins		
bmp2a<br>*bmp2b*	Bone morphogenetic protein 2a<br>Bone morphogenetic protein 2b	CRISPR/Cas9	Development	Partial deformation of ribs and vertebrae, massive death of embryos 12 hours after fertilization due to improper formation of somites, changes in the expression of genes that control skeletal development (*bmp2a, bmp2b, smad1, fgf4, runx2b, alp*)	Control of skeletal formation	[235]
nkx3.2	NK3 homeobox 2	CRISPR/Cas9	Development	Loss of the jaw joint, fusion of the occipital bones, defects of the Weberian apparatus, loss or deformation of bone elements derived from the basiventral cartilages of the vertebrae	Control of skeletal formation	[175]
mosmoa<br>*mosmob*	Modulator of smoothened a	CRISPR/Cas9	Development	Frontonasal hypoplasia, defects of the craniofacial skeleton in adult fish	Control of skull formation	[236]
scxa<br>*scxb*	transcription factor scleraxis	CRISPR/Cas9	Development	*scxb* mutants have a normal phenotype. *scxa* mutants have disorders in the formation of tendons and muscles in the cranial part of the body, abnormal mineralization and structure of rib bones and cartilage defects, impaired swimming movements, high mortality at the age of 2-5 weeks.	Control of tendons, bones, cartilage and muscle formation	[237]
cyp1b1	Cytochrome P450, family 1, subfamily B,	CRISPR/Cas9	Development	Microphthalmia and abnormal development of the jaw in F0,	Control of skull formation	[238]

	polypeptide 1			unilateral or bilateral craniofacial changes in F3, increased expression of genes that control processes associated with adhesion, extracellular matrix, cell growth and proliferation, lipid metabolism, inflammation		
hspa8	Heat shock protein 8	CRISPR/Cas9	Development	Malformations of the pharyngeal arches, pectoral fins, head, and eyes	Control of the development of the skeleton and organs of the cranial part of the body	[239]
stat3	Signal transducer and transcription activator 3	TALEN	Development	Scoliosis, susceptibility to spinal fractures, incomplete connection of bones, reduction of articular surfaces, decreased expression of immune response genes, collagen, genes that control skeletal development and fish growth	Control of spinal development, formation of an immune response	[240]
kif7	Kinesin family protein 7	CRISPR/Cas9	Development	Severe scoliosis, brain deformation, Reissner fibers, changes in the hedgehog signaling pathway	Control of skeletal formation by modulating the activity of the hedgehog signaling pathway	[241]
chrna1	Cholinergic receptor, nicotinic, $\alpha1$ (muscle)	CRISPR/Cas9	Development	Lack of ability to move	Control of fish movement ability	[294]
gdf5	Growth differentiation factor 5	CRISPR/Cas9	Development	Loss of the posterior radial bones of the pectoral fins, disorganization and reduction in the length of the bones of the median fins	Control of fin skeleton formation	[242]

	runx2b	Transcription factor 2b of the runx family (run-related transcription factors)	CRISPR/Cas9	Development	Loss of muscle bones (spicules) located in the myosepta of fish (connective septum between segments of skeletal muscles), without affecting the method of swimming, growth, bone mineral density, increased expression of the genes *tgf-β* (transforming growth factor), *bmp* (bone morphogenetic protein)	Control of spicule formation in myosepta	[243]
	pomc	proopiomelanocortin	CRISPR/Cas9	Growth	Increased body weight due to more intense muscle formation without signs of obesity. Reducing the feed conversion ratio (the ratio of the weight of feed used to the weight of the resulting product). Reducing cholesterol levels in tissues.	Formation of skeletal muscles	[259]
	ik	IK cytokine	CRISPR/Cas9	Growth	Decreased expression of genes responsible for skeletal muscle differentiation; some genes in this group exhibit incorrect pre-mRNA splicing. *myod1* expression is decreased and *pax7a* is increased. Disturbance of neuromuscular contacts, rapid muscle development, myoblasts form myotubes worse	Formation of skeletal muscles	[245]
	foxm1	Forkhead box M1	CRISPR/Cas9	Growth	Death of muscle fibers, activation and proliferation of muscle stem cells. High levels of Cas9 expression have been found to be toxic to muscles	Modulation of muscle response to fiber death	[210]

	dyrk1b	Dual-specificity tyrosine phosphorylation-regulated kinase 1B	CRISPR/Cas9	Growth	Reduced production of myosin heavy chain 1, underdevelopment of myotomes (skeletal muscle rudiments), which leads to the death of embryos. As part of the work, the 4e-bp1 protein with which *dyrk1b* interacts was identified. This protein enhances autophagy processes in muscle cells and modulates the effects of *dyrk1b*	Formation of skeletal muscles	[246]
	vcp	Valosin-containing protein	CRISPR/Cas9	Growth	Impaired functioning of cardiac and skeletal muscles, serious defects in the organization of myofibrils within muscle cells, the formation of foreign bodies in them, mitochondrial degeneration, accumulation of ubiquitinated proteins, which is likely due to improper functioning of proteasomes, increased levels of UPR/ER stress markers and signal transmission along the mTOR pathway, which indicates the activation of autophagy processes	Formation of muscles, maintaining protein homeostasis	[247]
	tpcn1	Two pore segment channel 1 (TPC1)	CRISPR/Cas9	Growth	Disruption of myoseptal connections (structures connecting the actin cytoskeleton of muscle cells with components of the extracellular matrix through transmembrane protein complexes), incorrect position of muscle cells relative to myosepta, separation of cells from myosepta.	Control of the movement of TPC1-lysosomes, membrane contacts, contractile activity of muscle cells	[248]

				Lysosomes, which contain the TPC1 protein in their membrane, are dynamically attached to the myoseptal junctions via microtubules. In the absence of *tpcn1* expression, lysosome transport is disrupted and β-dystroglycan, a key transmembrane component of the dystrophin-associated protein complex, is misdistributed		
greb1	Estrogen regulator of breast tumor growth 1	TALEN	Growth	Disturbances of convergent stretching in embryogenesis, developmental delay during gastrulation, secretory cells are reduced, including somatotropic, thyrotropic, lactotropic, gonadotropic, decreased production of growth hormones, thyrotropin, prolactin, follicle-stimulating, luteinizing, high embryo mortality	Control of gastrulation, formation of secretory cells, control of hormone synthesis	[261]
tshba<br><br>*tg*<br>*slc16a2*	βa subunit of thyrotropin<br>Thyroglobulin<br>Solute transporter family protein 2 16	TALEN<br>CRISPR/Cas9	Growth	In *tshba* mutants, an early change in the amount of the hormone T3 begins to occur, which leads to delayed development of the swim bladder, abnormal development of fins and scales, impaired transition from the larval stage to the juvenile stage, growth retardation, mating failure, and incorrect formation of secondary sexual characteristics. *tg* mutants exhibited growth retardation. The *td* and *slc16a2*	Control of the transition from the larval stage to the juvenile stage, the formation of secondary sexual characteristics	[262]

				mutants showed severe goiter		
duox	Double oxidase	CRISPR/Cas9	Growth	Hypothyroidism, growth retardation, goiter, infertility, absence of antennae, pigmentation defects, erythema in the eye area, torn fins, delayed scale formation. The normal phenotype was restored by treatment with thyroxine (T4)	Control of thyroid hormones	[263]
tpo	Thyroid peroxidase	CRISPR/Cas9	Growth	Slow growth, enlarged follicular cells in the thyroid gland, pigmentation defects, delayed development of scales, swim bladder defects. The normal phenotype was restored by treatment with T4 hormone. Increasing glucose levels in larvae by increasing glucagon expression and decreasing insulin expression	Control of thyroid development and glucose homeostasis	[66]
isl2a<br>*isl2b*	Insulin gene enhancer protein ISL-2 (ISL LIM homeobox 2)	CRISPR/Cas9	Growth	Hypoplasia of the thyroid gland, decreased levels of thyroid hormones, early mortality, sexual imbalance, disturbances in body structure during growth, a decrease in the number of pituitary cells, decreased expression of pituitary and thyroid hormone genes (*tshba, cga, tg*)	Control of thyroid and pituitary hormones<br>Control of pituitary cell differentiation	[117]
katnal2	Katanin p60 subunit A-like 2	CRISPR/Cas9	Growth	Delayed embryonic development, prolonged stages of convergence and elongation, decreased brain size and body length, decreased motor activity, increased activity	Control of embryogenesis, body size, behavior	[81]

				at night in larvae, increased anxiety behavior, and difficult social interactions		
smc5	Protein that maintains the structure of chromosome 5	CRISPR/Cas9	Growth	Microcephaly, shortening of the body, impaired glucose metabolism, triggering of apoptosis through activation of the p53 protein	Maintaining the stability of genetic elements and carbohydrate metabolism	[249]
stat5.1	Signal transducer and transcription activator 5b	CRISPR/Cas9	Growth	Reducing the body length and weight of the fish. More than 800 transcriptional targets have been identified for the gene under study, including genes for metabolic and signaling pathways and neuroactive interactions	Control of body length and weight of fish	[250]
lepr	Leptin receptor	CRISPR/Cas9	Growth	72 hours after fertilization, when zebrafish pass from the embryonic stage of development to the larval stages, the mutants exhibit reduced oxygen consumption and a reduced metabolic rate. Changes in mutant larvae in the expression of genes that control appetite and energy metabolism (*cart*, *npy*, *agrp*, *mc4r*), perception by the senses (*casr*, *t1r1*, *t1r3*, *t1r2-1*, *t1r2-2*, *pept1a*, *pept1b*), digestion (*cck*, *pyy*, *try*, *ct*, and *amy*)	Control of processes related to nutrient acquisition and digestion	[264]
lepa<br>*lepb*<br>*lepr*	Leptin<br>Leptin<br>Leptin receptor	CRISPR/Cas9	Growth	The development of obesity, gonadal development, the process of puberty, and sex formation were assessed. No mutant phenotype was detected	In fish, unlike mammals, the leptin system does not play a significant role in the regulation of	[266]

					growth and reproduction (puberty)	
lepb	Leptin b	CRISPR/Cas9	Growth	Increased fish body weight and length, accumulation of visceral fat, increased blood glucose levels, glomerular hypertrophy, thickening of the glomerular basement membrane, which is a symptom of diabetic nephropathy	Control of glucose metabolism, maintenance of normal adipocytes, weight and growth of fish	[265]
rfx6	Regulatory factor X, 6	CRISPR/Cas9	Growth	Symptoms of diabetes mellitus, islets of Langerhans not forming in the pancreas, decreased amount of insulin. It has been shown in mice that rfx6 interacts with translation initiation factors and controls insulin translation	Initiation of insulin translation	[267]
akr1a1a	Aldo-ketoreductase family 1, member A1a	CRISPR/Cas9	Growth	Accumulation of acrolein in larvae and in the liver of adult mutants, impaired glucose homeostasis, impaired glucose tolerance, signs of diabetic retinopathy, nephropathy	Controlling the excretion of acrolein from the body, maintaining glucose homeostasis	[268]
igf1	Insulin-like growth factor 1	CRISPR/Cas9	Growth	A decrease in the size of animals, an increase in the expression level of insulin, insulin-like growth factors 2 and 3. In females, an increase in the expression of the genes *gh1* (growth hormone), *pepck* (phosphenolpyruvate carbooxykinase), *erk1/2* (phosphorylated kinase regulated by extracellular signal), a decrease in the ratio lactate/pyruvate in the	In males – activation of signal transmission along the AKT/mTOR pathway, in females – control of glucose metabolism	[269]

				liver, hyperglycemia. In males, fatty liver, decreased levels of ribosomal kinase S6		
rreb1a *rreb1b*	Ras responsive element binding protein 1a	CRISPR/Cas9	Growth	Decreased insulin production, decreased amount of insulin in cells, decreased insulin production with prolonged stimulation of the pancreas, improper differentiation of proendocrine cells	Control of insulin synthesis, endocrine cell differentiation	[270]
glo2	Glyoxylase 2	CRISPR/Cas9	Growth	Accumulation of SD-lactoylglutathione in larvae with preservation of normal glutathione metabolism. In the liver of adult fish, hexose concentration decreased, and decreased activation of P70-S6 kinase was observed after food intake	Control of cell energy metabolism	[271]
nur77	Nuclear receptor subfamily 4, group A, member 1	CRISPR/Cas9	Growth	Increased levels of total cholesterol, triglycerides, glucose, changes in the amino acid profile, changes in the expression of genes responsible for the metabolism of amino acids, fats, carbohydrates. The reason for the increase in glucose levels is an imbalance of glycolysis and gluconeogenesis, a decrease in insulin synthesis by β-cells, a decrease in the expression of *insb* (preproinsulin b) in them, and a decrease in their number	Control of fat and glucose metabolism, insulin synthesis	[272]
pparγ	Peroxisome proliferator-activated receptor γ	CRISPR/Cas9	Growth	Hyalinization of the liver, the appearance of compaction foci in it; when fed with food with a high fat content, there was no	Control of the activity of enzymes involved in fat metabolism	[273]

				accumulation of visceral fat, phosphorylation of serine/threonine protein kinase TOR, ribosomal protein kinase S6, and mitogen-activated protein kinase 14A		
phlpp1	PH domain and leucine-rich protein repeat phosphatase 1	CRISPR/Cas9	Growth	Reducing the accumulation of fats in the walls of blood vessels, reducing total cholesterol and triglycerides	Controlling fat accumulation	[274]
cygb1	Cytoglobin	CRISPR/Cas9	Growth	Weight loss, activation of immune and cell cycle regulators in the liver (*tp53*), changes in the transcription of genes responsible for fat metabolism and transport, antioxidant protection, maintaining iron balance	Proposed functions: anti-inflammatory and cell protective factor in the liver, participation in the metabolism of fats, iron and signal transduction through the ROS-dependent pathway	[275]
elovl8a	ELOVL fatty acid elongase 8a	CRISPR/Cas9	Growth	Impaired biosynthesis of long-chain polyunsaturated acids from C18:2n-6, C18:3n-3 to C20:2n-6, C20:3n-3, as well as C18:0 and C20:1 acids	Control of the synthesis of long-chain polyunsaturated fatty acids	[276]
fads2	Desaturase with Δ6 and Δ5 activity	CRISPR/Cas9	Growth	Violation of the biosynthesis of long-chain polyunsaturated ω6 and ω3 fatty acids led to blocking of the conversion of linoleic acid into γ-linolenic acid, and *α*-linolenic acid into stearidonic acid, due to which the caviar was formed defective	Control of the synthesis of long-chain polyunsaturated fatty acids	[277]

t1r1	Taste receptor 1 type 1	CRISPR/Cas9	Growth	There is no sensitivity to alanine, reduced demands on food, they eat duckweed and vegetable protein. A plant-based diet suppressed the expression of the orexigenic gene (encoding hunger peptides) *npy* and increased the expression of the anorexigenic gene *pomca*. When switched to a standard diet, the expression profile of *npy* and *pomca* in mutants remained altered compared to the wild type	Control of eating behavior, absorption of amino acids	[280]
acvr2aa *acvr2ba*	Activin A receptor, type II	CRISPR/Cas9	Growth	Double mutants *acvr2a–/–b–/–* show hypertrophy of muscle fibers, increased muscle growth, body weight, and ventricle area in heart	Negative regulator of skeletal muscle mass	[260]
slc45a2	Solute transporter family protein 2 45	CRISPR/Cas9	Pigmentation	Reduced pigmentation	Control of fish body color	[294]
pcdh10a and pcdh10b	Protocadherin 10	TALEN and CRISPR/Cas9	Pigmentation	Deletion of *pcdh10a* results in abnormal migration of melanophore precursors due to increased adhesion and loss of cell-cell contact with other migrating neural crest cells, a compensatory increase in *pcdh10b* expression. Deletion of *pcdh10b* resulted in somite defects. Double knockout of *pcdh10a* and *pcdh10b* resulted in increased abnormal migration of melanophore precursors and embryonic lethality	Control of migration of melanophore precursors to the site of differentiation	[282]

tg<br>*scarb1*<br>*tyr*<br>*thraa*<br>*thrab*<br>*thrb*	Thyroglobulin<br>Receptor-HDL 1 class B<br>Tyrosinase<br>α-thyroid hormone receptor<br>α-thyroid hormone receptor<br>β-thyroid hormone receptor	CRISPR/Cas9	Pigmentation	Fish without thyroid hormones have twice as many melanophores as wild type and no xanthophores. Mutants in the thyroid hormone receptor genes have a wild-type phenotype. Double mutants without thyroid hormones and without receptors for them formed xanthophores. The number of melanophores in such fish was comparable to the wild type	Thyroid hormones direct melanophores to final differentiation and stimulate xanthophores to accumulate carotinoids.<br>Thyroid hormone receptors repress pigment cell differentiation programs under conditions of thyroid hormone deficiency	[283]
bmp7b	Bone morphogenetic protein 7	CRISPR/Cas9	Pigmentation	Knockout of *bmp7b* led to growth retardation, increased melanin synthesis in the skin and eyes, forotoronic induction, eating disorders, photo. Transcriptomic analysis of such fish showed an increase in the expression of genes that control melatonin synthesis (*wnt7ba* and *gna14*), disruption of the work of genes whose proteins are potentially involved in the cell response to photon excitation (*gs9a, rgs9b, rcvrn2, guca1d, grk1b, opn1mw4, gc2*)	Control of melatonin synthesis, modulation of genes that mediate cell response to photons	[223]
pax3a<br>*pax3b*<br>*pax7a*<br>*pax7b*	Paired box proteins	TALEN, CRISPR/Cas9	Pigmentation	Pax3a mutants had a delay in xanthophore formation and decreased expression of *pax7a* and *b*. In *pax7a* double mutants; *pax7b*	Formation of xanthophores, melanophores<br>The *pax3* gene	[285]

				xanthophores were completely absent. When *pax7* was knocked out, the number of melanophores was increased	activates the expression of the *pax7* gene	
plin6	Perilipin	TALEN	Pigmentation	Decreased ability of carotenoid droplets to accumulate carotenoids; carotenoid droplets do not merge with carotenoid bodies in xanthophores	Control of carotenoid accumulation in xanthophores	[286]
scarb1 715delA *scarb1 715_716insGG*	Scavenger receptor class B1 (scavenger receptors)	CRISPR/Cas9	Pigmentation	Impaired accumulation of carotenoids and, accordingly, pigmentation, decreased fertility, decreased survival of larvae	Control of carotenoid accumulation, reproduction	[287]
alk<br>*ltk*<br>*alkal1 (aug-α1, aug-α2)*<br>*alkal2(aug-β)*	Anaplastic lymphoma kinase<br>Leukocyte tyrosine kinase<br>Anaplastic lymphoma kinase and leukocyte tyrosine kinase ligand 1<br>Anaplastic lymphoma kinase and leukocyte tyrosine kinase ligand 2a	CRISPR/Cas9	Pigmentation	Impaired formation of iridophores and pigmentation of the skin and eyes in embryos and adult fish. Knockout fish for ligand genes exhibit the same iridophore disturbance as *ltk* mutants.	The *ltk, aug-α1, aug-α2, aug-β* genes, but not *alk*, control the formation of iridophores and the coloration of the skin and body of fish	[288]
edn3a<br>*edn3b*<br>*ednrb1a*	Endothelin-3<br>b1a endothelin receptor	CRISPR/Cas9	Pigmentation	The *edn3a* mutants were almost identical to the wild type, but they had fewer iridophores on the abdomen, which caused it to have a pinkish tint. In edn3b mutants, the iridophores and melanophores of the hypodermis were largely lost, and melanophore stripes were not formed. Both staining defects	Control of species-specific proliferation of iridophores<br>The ednrb1a gene ensures the interaction of ligands *edn3a* and *edn3b*	[289]

				were observed in the *edn3a* and *edn3b* double mutants. The *ednrb1a* mutants were colored like *edn3a* and *edn3b* knockout fish. The spotted danio *Danio nigrofasciatus* has the same coloration as the *edn3b* mutant		
sox5<br><br><br><br>*sox10*	SRY-related (sex-determining region on Y chromosome) transcription factor 5<br>SRY-related transcription factor 10	TALEN, CRISPR/Cas9, TILLING	Pigmentation	*sox10* mutants do not develop melanophores, iridophores, or xanthophores. Knockout of *sox5* in *sox10* mutants partially restored normal differentiation of pigment cells	The *sox10* gene controls the differentiation of melanophores, iridophores, xanthophores<br>The *sox5* gene represses the differentiation of pigment cells under the influence of *sox10*	[284]
mc1r	Melanocortin receptor 1	CRISPR/Cas9	Pigmentation	Absence of dorso-ventral countershading, more melanophores and iridophores on the back, fewer iridophores on the abdomen than in the wild type, lighter body coloration	Formation of dorsoventral countershading	[295]
asip1	Agouti signaling protein	CRISPR/Cas9	Pigmentation	Absence of dorsoventral countershading due to changes in the number of pigment cells	Formation of dorsoventral countershading	[296]
cyp19a1a	Aromatase	TALEN<br>CRISPR/Cas9	Sex	All knockout fish were male, but they retained immature oocytes and oocyte-like germ cells	Formation of the female gender	[299]

	cyp19a1a	Aromatase	TALEN	Sex	Females did not produce estrogen, resulting in sex reversal	Formation of the female gender	[300]
	cyp19a1a *cyp19a1b*	Aromatase	TALEN; CRISPR/Cas9	Sex	Homozygotes for the *cyp19a1a* gene knockout variant and heterozygotes for the *cyp19a1a* and *cyp19a1b* knockout variants were all males. In homozygotes with a knockout of the *cyp19a1a* gene, sex formation was delayed, but treatment with 17β-estradiol partially restored the normal phenotype. The expression of genes regulating sex formation decreased	The *cyp19a1a* gene controls the formation of female sex	[301]
	cyp19a1a *dmrt1*	Aromatase doublesex and mab-3 related transcription factor 1	CRISPR/Cas9	Sex	All *cyp19a1a* knockout fish were males. Among the dmrt1 mutants, there were more females than males. Mutants for both genes were also males, but in them, despite the absence of estrogen, follicles formed normally up to the pre-vitellogenin stage. In the presence of aromatase, the *dmrt1* gene was repressed by estrogen receptors	Formation of the female sex, control of folliculogenesis	[302]
	pgr	Progesterone receptor	TALEN	Sex	Sterility of homozygous mutant females. Heterosiotes formed normal eggs, but ovulation did not occur. Heterozygous males were fertile	Control of signal transmission between follicular and germ cells	[305]
	fshb	β-subunit of follicle-stimulating hormone β-subunit of	TALEN	Sex	Mutants for the *fshb* gene showed a delay in the development of ovaries and testes, but ultimately	The *fsh* gene controls the formation of the	[303]

lhb	luteinizing hormone			the individuals caught up with the wild phenotype and became fertile. Some females became males. Disruption of the *lhb* gene resulted in normal gonadal development in both males and females, but females were infertile due to impaired oocyte maturation. The double mutants were all males and their testes formation was delayed	female sex The *lhb* gene controls oocyte maturation in females	
fshr *lhcgr*	Follicle stimulating hormone receptor Luteinizing hormone receptor	TALEN	Sex	In *fshr* mutant females, follicle activation does not occur; oocytes are delayed in the transition from the growth stage to the previtellogenin stage. Later, such females turned into fertile males. In males, there was a delay in spermatogenesis, which was later compensated. The mutant phenotype for the *lhcgr* gene was not expressed. Double mutants for the studied genes were sterile males	The fshr gene *controls* the activation of follicles and the formation of the female sex	[304]
gnrh3	Gonadotropin-releasing hormone 3	TALEN	Sex	It has previously been shown that the absence of cells that do not express gonadotropin-releasing hormone 3 leads to infertility. In this study, normal gametogenesis and reproductive ability and fertility were observed in mutant fish, which indicates the presence of compensatory mechanisms that arise at the early stages of	Control of gonad formation	[374]

				differentiation of *gnrh* neurons		
gsdf *(TGFB family)*	Gonadal soma derived factor (Transforming growth factor β family)	TALEN	Sex	Long-term existence of gonads at the bipotential stage of development, subsequently in females - the development of single oocytes, sterility, immature follicles, lipid accumulation, decreased production of aromatase, *gata4*, insulin receptor, estrogen receptor and genes for lipid metabolism, vitellogenin and steroid biosynthesis. Androgen content is increased. The phenotype mimics human polycystic ovary syndrome, suggesting a role for the related signaling molecule TGFB in the etiology of the syndrome. Mutant males are fertile	Control of follicle maturation	[306]
esr2a<br><br>*esr2b*<br><br>*esr1*	Estrogen receptor βII<br>Estrogen receptor βI<br>Estrogen receptor α	CRISPR/Cas9	Sex	Mutants for any one of the genes studied showed a normal phenotype, which indicates a redundancy of estrogen receptor varieties. Viable eggs in *esr2a* mutant females had an enlarged and deformed chorion; larvae hatched from such eggs earlier than in the wild type. In female *esr2a* and *esr2b* double mutants and triple mutants, folliculogenesis was arrested at previtellogenin stage II. Subsequently, such females became males. Males developed a normal phenotype	Control of folliculogenesis, maintenance of female sex in adulthood	[307]

	sox3	SRY-related transcription factor 3	CRISPR/Cas9	Sex	Delayed development of follicles, decreased fertility in females, triggered apoptosis of oocytes and somatic cells of follicles, suppression of aromatase expression and decreased production of 17β-estradiol.	Control of follicle development and expression of female sex hormones	[308]
	inhbaa<br><br>*inhbab*<br><br>*inhbb*	βAa subunit of inhibin<br>βAb subunit of inhibin<br>βB subunit of inhibin	CRISPR/Cas9	Sex	Double mutants for *inhbaa, inhbab* had a high percentage of mortality after hatching from the eggs, they had increased expression of the *fshb, cyp19a1a* (aromatase) genes, females had a delay in the development of follicles, hypertrophy of granulosa cells, tissue fibrosis, males demonstrated normal spermatogenesis and fertility. In females with *inhbaa* knockout, follicular defects accumulated, with folliculogenesis completely stopped; tumors and cysts began to appear in the ovaries over time. In *inhbb* mutants of both sexes, germ cells developed normally, but immature follicles accumulated in females, which led to a decrease in their fertility	Control of folliculogenesis, steroid synthesis	[309]
	cyp17a1	17-α-hydroxylase (Cytochorem P450, family 17, subfamily A, polypeptide 1)	TALEN	Sex	Increased expression of the sox9a gene, decreased expression of the *amh* gene, which led to insufficient levels of testosterone and 11-ketotestosterone in the blood plasma of the fish, the	Participation in the signaling pathway of androgen synthesis	[311]

				development of male sex in all offspring, and disruption of mating behavior		
cyp17a1	17-α-hydroxylase (Cytochorem P450, family 17, subfamily A, polypeptide 1)	TALEN	Sex	Males have low levels of androgen in plasma, testosterone and 11-ketotestosterone in the brain, and the male pattern of behavior during the mating season is disrupted. Treatment of such males with 11-ketotestosterone restores secondary sexual characteristics and mating behavior. The gene knockout alters the expression of 358 genes compared to controls, including aromatase (*cyp19a1b*), progesterone receptor (*pgr*), deiodinase (*dio2*), insulin-like growth factor 1 (*igf1*), and others	Regulates male mating behavior and androgen-mediated signaling	[312]
cyp11c1	11-β-hydroxylase (Cytochorem P450, family 11, subfamily C, polypeptide 1)	CRISPR/Cas9	Sex	In males, decrease in the size of the genital papilla, impaired mating behavior, normal gametes, a delay and prolonged transition of the juvenile ovary to the testis, a decrease in the expression of the genes *insl3, cyp17a1, amh, dmrt1*. In females, spawning is inhibited and the germinal vesicles are destroyed	Control of testes maturation, Leydig cell development, spermatogenesis	[313]
ar	Androgen receptor	CRISPR/Cas9	Sex	An increase in the proportion of females among mutant offspring, the formation of secondary female sexual characteristics in males, impaired spermatogenesis,	Control of testes development and egg maturation	[314]

				impaired oocyte maturation, impaired regulation of the synthesis of sex hormones		
ar	Androgen receptor	TALEN	Sex	Reduced testicular size, sperm sterility, increased expression of steroid genes, decreased levels of estradiol and 11-ketotestoserone	Monitoring the development of testes and the level of sex hormones	[316]
ar	Androgen receptor	CRISPR/Cas9	Sex	In mutant males, development of ovaries and female secondary sexual characteristics was observed, in a small part of males, secondary sexual characteristics of females, abnormal formation of cysts, absence of a central pool of sperm and seminiferous tubules, inability because of this to secrete sperm, increased expression of the *cyp19a1a, foxl2a* genes in the testes (profile characteristic of ovaries). In mutant females, the development of oocytes stopped at stage I, and the expression of the *amh* and *dmtr1* genes increased in the ovaries (a profile characteristic of the testes)	Determination of sex, development of testes, maturation of female germ cells	[315]
amh<br><br>*fshb*<br><br><br><br>*fshr*	Anti-Mullerian hormone<br>β-subunit of follicle-stimulating hormone<br>Follicle stimulating hormone receptor<br>Luteinizing hormone receptor<br>Bone morphogenetic	CRISPR/Cas9	Sex	Gonadal hypertrophy, hyperproliferation of ovarian cells, delayed gametogenesis, decreased *fshb* expression in *amh* mutants. Normal gonads in *amh*, *fshr*, *lhcgr* mutants. *bmpr2a* mutants recapitulate the phenotype of *amh* mutants.	The *amh* gene modulates gonadotropin-mediated signal transduction, proliferation and differentiation of germ cells	[317]

lhcgr<br><br><br>*bmpr2a*<br><br><br><br><br>*bmpr2b*	protein II a (serine/threonine kinase)<br>Bone morphogenetic protein II b (serine/threonine kinase)					
amh1	Anti-Müllerian hormone	CRISPR/Cas9	Sex	Among *amh* mutants, females predominated; gonadal hypertrophy was observed due to proliferation and incomplete differentiation of germ cells	Maintaining a balance between cell proliferation and differentiation	[318]
wnt4a	Wingless-type family member 4	CRISPR/Cas9	Sex	Predominance of males among mutant offspring. Both males and females are sterile due to malformations of the reproductive tract	Formation of the female sex, control of the development of the reproductive ducts	[319]
myoc	Myocycline	CRISPR/Cas9	Sex	All mutants were males, increasing the expression of genes responsible for the formation of male sex	Control of ovarian differentiation	[320]
egf<br><br>*egfra*<br>*egfrb*	Epidermal growth factor<br>Epidermal growth factor receptor	CRISPR/Cas9	Sex	In *egf* mutants, the number of eggs capable of fertilization decreased. In *egfra* mutants, follicle activation did not occur, which led to infertility in females and subsequently to sex reversal. The males were fertile. Changes in the expression of genes involved in oxygen metabolism, transcription, membrane transport, steroid synthesis, and immune response were also observed. *egfrb* mutants	Control of early folliculogenesis	[321]

				formed a normal phenotype		
nobox	Newborn oocyte-specific homeobox protein	CRISPR/Cas9	Sex	The developing ovaries reached the stage of primary growth, the follicles stopped at the cyst stage and did not become functional, the females turned into males. The knockout did not affect the development of testes, spermatogenesis, or spawning behavior in males	Ovarian formation	[322]
scg2a<br>*scg2b*	Secretogranin II a<br>Secretogranin II b	TALEN	Sex	Disorders of sexual behavior, ovulation, spawning. The normal phenotype was partially restored by secretoneurin injections	Ovulation control	[325]
parn	Poly(A)-specific ribonuclease	CRISPR/Cas9	Sex	All mutants were fertile males; underdeveloped oocytes were found in the gonads	Control of oogenesis	[323]
avp	Vasotocin	CRISPR/Cas9	Sex	A decrease in the number of fertilized eggs in a pair of mutant fish, a disruption in the mating behavior of the female at the time of spawning, a decrease in the number and disruption of oocyte maturation, a decrease in the level of prostaglandin F2α. Treatment with vasotocin did not restore the normal phenotype. Males showed a normal phenotype	Control of oocyte maturation	[326]
ambra1b	Beklin 1	CRISPR/Cas9	Sex	Sex cells did not develop; the offspring were only males. The normal phenotype was restored by injection of human ambra1 ambra1b mRNA, but was not restored by injection of human	Preservation of germ cells due to binding of the Ambra1 protein to the CUL4-DDB1 complex, control of	[324]

				ambra1 with an altered region encoding the CUL4-DDB1 binding region	mating behavior	
bmp15	Bone morphogenetic protein 15	TALEN	Sex	Females with a disrupted *bmp15* gene demonstrated an arrest in oogenesis, and sex change occurred at the middle or late stages of larval development	Formation of the female gender	[300]
bmp15<br><br>*inha*<br>*inhbaa*	Bone morphogenetic protein 15<br>Inhibin<br>βAa subunit of inhibin/activin	CRISPR/Cas9	Sex	In mutant females for the *bmp15* gene, there was a delay in the development of follicles, their arrest at the previtellogenin stage, no yolk formation occurred, a delay in puberty, and sex reversal with the formation of secondary sexual characteristics were also observed. In *bmp15* and *inha* double mutants, follicle development progressed to the mid-vitellogenin stage, and sex reversal did not occur. In triple mutants for *bmp1, inha, inhbaa*, yolk granules again disappeared. Estradiol and vitellogenin levels were reduced in *bmp15* mutants, restored in double mutants, and decreased again in triple mutants. Treatment with estradiol restored the normal phenotype in *bmp15* mutants	Bmp1 interacts with the activin/inhibin system, controls estradiol levels and vitellogenin synthesis	[310]
dmrt1	Doublesex and mab-3 related transcription factor 1	CRISPR/Cas9	Sex	Most mutants are fertile females, apparently due to sex reversal in a large proportion of males. Males are sterile, intersex gonads with	Transcriptional regulation of sex, control of testes development	[328]

				intact oocytes and dysmorphic sperm. The expression of the *amh* (anti-Müllerian hormone) and *foxl2* (forkhead box L2) genes, which are important for the formation of male and female sex, respectively, is disrupted. Females have normal gonads		
dmrt1	Doublesex and mab-3 related transcription factor 1	CRISPR/Cas9	Sex	In *dmrt1* mutants, severe disturbances in the differentiation of male germ cells were revealed, the process of cell renewal did not occur, and apoptosis was triggered in many	Maintaining vitality, renewal, differentiation of male germ cells	[318]
mettl3	Methyltransferase-3-like enzyme	TALEN	Sex	In mammals and plants, a homozygous *mettl3* mutation is lethal. A viable mutant was obtained for zebrafish. In such females, the oocytes stopped at an early stage of development. In males, sperm maturation was blocked, and the levels of the hormones 11-ketotestosterone and 17β-estradiol were reduced	Control of germ cell maturation	[331]
dnd	dead end	CRISPR/Cas9	Sex	Homozygotes with a knockout variant of the gene did not form gametes; in adulthood, all mutants became males with intact mating behavior, but they did not produce sperm. Heterozygotes did not differ from the wild type	Control of germ cell maturation	[332]
vtg1, 3, 4, 5	Vitellogenin	CRISPR/Cas9	Sex	Increased expression of the *vtg7* gene due to an unknown mechanism for maintaining	Control of egg formation, embryogenesis,	[334]

				vitellogenin homeostasis. In *vtg1* knockout females, the number of eggs per spawning increased. In *vtg3* knockout females, egg fertility decreased. In F1, the mortality of eggs and embryos increased, the proportion of hatched larvae decreased, the larvae showed swelling of the pericardium and yolk sac, lordosis of the spine, impaired motor and feeding activity. At later stages of development, all descendants of mutant females for *vtg1* and almost all for *vtg3* died	skeletal formation, lethality in late larval stages	
stm	Starmaker protein	CRISPR/Cas9	Sex	Low survival rate of fish, incorrect formation of otoliths in the inner ear (but the swimming method remained normal), incorrect formation of fibrous pineal structures in the shell of the eggs	Control of the formation of the egg shell and otoliths of the inner ear	[335]
gpr56	adhesion G protein-coupled receptor G1	CRISPR/Cas9	Disease resistance	Mismatch in the expression of genes involved in immune reactions, decreased expression of genes for digestive enzymes. The mutants had increased motor activity and reacted vividly to cold and other irritants	Control of immunity, digestion and excitability	[337]
socs3b	suppressor of cytokine signaling 3b	CRISPR/Cas9	Disease resistance	The mutants have an increased number of neutrophils, but they are not functional. The number of macrophages does not change, but their activity is increased. The survival rate of mutants in	Control of distribution and activity of neutrophils and macrophages	[194]

				adulthood is reduced, there is eye pathology associated with infiltration of this area by neutrophils and macrophages, and impaired coordination of immune cells in other tissues		
mitfa	melanocyte inducing transcription factor a	CRISPR/Cas9	Disease resistance	A decrease in the amount of melanin, in the presence of *Edwardsiella tarda* and biphenazate - a decrease in the number of macrophages, an increase in the expression of immune response genes except *lr4* and *rela*	Probable negative regulator of immune response	[195]
CD18	β subunit of β2 integrins	CRISPR/Cas9	Disease resistance	The mutants exhibit an increased number of neutrophils, but they do not migrate to sites of inflammation	Control of neutrophil migration	[154]
lcp1	lymphocyte cytosolic protein 1 (L-plastin)	CRISPR/Cas9	Disease resistance	Reduced survival into adulthood, presumably due to the negative impact of knockout on the immune system. Macrophages are stellate in shape and move slowly and in an undirected manner, presumably due to a disruption in the formation of actin filaments	Control of macrophage morphology and migration	[159]
fam76b	family with sequence similarity 76 member B	CRISPR/Cas9	Disease resistance	Reduction in the size of the thymus, intense inappropriate inflammation, increased mortality of mutants	Regulation of hematopoiesis, immunity, inflammation	[338]
stat5.1	signal transducer and activator of transcription 5	CRISPR/Cas9	Disease resistance	Decreased embryonic T-lymphopoiesis, growth retardation, obesity	Control of lymphopoiesis, growth	[196]
viperin	viperin	CRISPR/Cas9	Disease resistance	The mutants were infected with hemorrhagic septicemia virus	Resistance to viral diseases, in	[197]

				(VHSV). Viral NP protein expression was increased compared to wild type. Expression of interferon genes φ1 and 3 was also reduced. Increased mortality of mutants was observed	particular viral hemorrhagic septicemia	
rrm1	ribonucleotide reductase M1 polypeptide	CRISPR/Cas9	Disease resistance	Gene knockout mutants are more susceptible to NNV	NNV resistance	[339]
prmt7	Protein arginine methyltransferase 7	CRISPR/Cas9	Disease resistance	Gene knockout mutants are more resistant to SVCV and GCRV infection	Susceptibility to SVCV and GCRV infection	[340]
sting	stimulator of interferon response cGAMP interactor	CRISPR/Cas9	Disease resistance	In seven-day-old mutant larvae, in the presence of the bacterium *Edwardsiella piscicida*, the expression of genes in the cyclic GMP-AMP synthase – Sting signaling pathway was first activated. In adult animals, the expression of these genes was significantly suppressed. Increased mortality of adult mutants was observed.	Resistance to the bacterium *Edwardsiella piscicida*	[199]
tnf-α1	Tumor necrosis factor α type I isoform	CRISPR/Cas9	Disease resistance	Increased mortality in the presence of *Edwardsiella piscicida*. Changes in the expression of genes regulated by *tnf-α1*.	Resistance to the bacterium *Edwardsiella piscicida*	[342]
crp	C-reactive protein	CRISPR/Cas9	Disease resistance	A more severe course of pneumococcal infection than in the wild type.	Resistance to the bacterium *Streptococcus pneumoniae*	[200]
itln3	intelectin 3	CRISPR/Cas9	Disease resistance	No distinct mutant phenotype	No effect on mycobacterial resistance or	[62]

						susceptibility	
	cxcr3.3<br><br><br>*cxcr3.2*	chemokine (C-X-C motif) receptor 3, tandem duplicate 3<br>chemokine (C-X-C motif) receptor 3, tandem duplicate 2	CRISPR/Cas9	Disease resistance	*Cxcr3.3* mutants are more susceptible to mycobacteria than the wild type. Macrophages in such mutants are more mobile, more easily activated, and more actively reach sites of inflammation. *cxcr3.2* mutants are more resistant to mycobacteria than the wild type. *Cxcr3.3* is an atypical chemokine receptor that binds to common ligands, resulting in upregulation of the *cxcr3.2* signaling pathway.	The *cxc3.3* gene mediates resistance to mycobacteria, *cxc3.2* mediates susceptibility.<br>*cxc3.3* limits the mobility and activation of macrophages and enhances signaling along the *cxc3.2* pathway.	[343]
**Rainbow trout**	*igfbp-2b1*<br>*igfbp-2b2*<br>*igfbp-2b2*	Insulin-like growth factor binding protein	CRISPR/Cas9	Growth	Reduced concentration of IGF-I (insulin-like growth factor) in the blood plasma, decreased weight and growth of fish. At 10 and 12 months after hatching, the growth delay was compensated to some extent	Fish growth control	[251]
	igfbp-2b1<br>*igfbp-2b2*	Insulin-like growth factor binding protein	CRISPR/Cas9	Growth	A decrease in plasma IGFBP-2 by 83% leads to a decrease in the amount of IGF-I by 35%. In the absence of food, due to changes in the expression of the *igfbp* gene, the inhibition of IGF-I production is reduced, since its amount in plasma is already small due to hunger	Monitoring the level of IGF-I in the blood depending on the amount of food	[252]
	lepra1<br>*lepra2*	Leptin receptor	CRISPR/Cas9	Growth	Hyperphagic phenotype, increased body weight, rapid growth, increased glycogen content in muscles, leptin in blood	Hunger control	[93]

				plasma, increased expression of the *pomc-b* gene (proopiomelanocortin).		
	sdY	sexually dimorphic on the Y chromosome	ZFN	Sex	In mutant males with a gene knockout, the gonads became ovaries. Transgenic females with increased expression of this gene developed testes. The gene has been identified for the first time	Formation of testes	[329]
	sdY	sexually dimorphic on the Y chromosome	ZFN	Sex	In F1, the mutants developed ovaries. One of the mutations resulted in the loss of leucine at position 43 of the protein	Formation of the male gender	[330]
	dnd	dead end	CRISPR/Cas9	Sex	Initially, the mutants formed the same number of primordial germ cells as the wild type, but then their number began to decrease. Subsequently, such fish were used as recipients of wild-type germ cells. They developed only donor germ cells	Formation of germ cells	[333]
**Atlantic salmon**	*fads2* *Δ6abc/5Mt* *Δ6bcMt*	Fatty acid desaturases	CRISPR/Cas9	Growth	In *fads2* gene mutants on a diet low in double-chain polyunsaturated fatty acids (DC-PUFAs), the production of omega-3 fatty acids 20:5n-3 and 22:6n-3 decreased and the production of 18:2n-6 and 18:3n- increased. 6. In the same mutants fed a diet high in LC-PUFA, the omega-3 composition was the opposite. In mutants for the desaturase gene *Δ6abc/5Mt*, a disruption in the synthesis of acid 22:6n-3 and increased production	Regulation of absorption and synthesis of fatty acids	[278,279]

				of 18:2n-6, 18:3n-3, 20:4n-3 were observed. On a diet low in LC-PUFA, these fish showed increased expression in the liver and cecum of fat synthesis gene transcription factors and sterol regulatory element binding protein 1 (*srebp-1* gene). Double mutants for the desaturase genes *Δ6abc/5Mt* and *Δ6bcMt* showed increased production of acids 20:2n-6 and 20:3n-3 on a diet low in LC-PUFA. The composition of dietary LC-PUFAs influences the expression of the srebp-1 gene, which in turn affects the overall LC-PUFA composition of salmon. *srebp-2* is the main regulator of endogenous synthesis of polyunsaturated fatty acids in Atlantic salmon.		
tyr<br>*slc45a2*	Tyrosinase<br>Solute transporter family protein 2 45	CRISPR/Cas9	Pigmentation	*slc45a2* mutants either completely lost pigmentation or retained only a few spots of pigment. In double mutants, pigmentation was preserved to varying degrees, i.e. from complete loss to close to the wild type	Pigmentation control	[23]
dnd<br>*alb*	dead end<br>albinism	CRISPR/Cas9	Sex	The ovaries of mutant females contained many eggs at the previtellogenin stage of maturation. The transition to the next stage did not occur, since cortical alveoli were not formed. In	Control of gonad maturation, control of the formation of the somatic compartment of the ovaries	[281]

					males, maturation of the testes also did not occur. Albinism was the mark of a successful knockout		
**Common carp**	*mstnba*	Myostatin	TALEN CRISPR/Cas9	Growth	Mutants for the *mstnba* gene weighed more and were larger than wild-type fish due to an increase in body length and muscle number (number of fibers, their hypertrophy and hyperplasia.	Limiting muscle hypertrophy and hyperplasia	[24]
	mstn	Myostatin	CRISPR/Cas9	Growth	Increase in length, thickness and body weight, increase in muscle mass due to hypertrophy and hyperplasia of muscle fibers, increased expression of myogenic differentiation factors, myogenic factor 5, myogenin	Controlling muscle growth	[253]
	sp7a	Transcription factor sp7	TALEN CRISPR/Cas9	Growth	*sp7a* mutants were smaller and lighter than wild-type animals. They had insufficiency of the gills, upper jaw, deflection of the back, shortening of the intermuscular bones. Parameters such as bone volume, bone surface area, and their ratio to tissue volume were reduced. The teeth were smaller and positioned incorrectly	Control of normal bone development	[24]
	cyp17a1	17-α-hydroxylase<br><br>(Cytochorem P450, family 17, subfamily A, polypeptide 1)	CRISPR/Cas9	Sex	All mutants were fertile males with normal testes, spermatogenesis, and functional sperm. Such males were crossed with wild-type females. In F1, all offspring were heterozygous females, with normal ovaries and increased body weight	Formation of the female gender	[7]

**Oujiang carp**	*asip1*<br>*asip2*	Agouti signaling protein (melanocortin antagonist in the melatonin synthesis signaling pathway)	CRISPR/Cas9	Pigmentation	Red carp with black spots were edited. In the mutants, the black spots disappeared, and the melanophores were randomly distributed over the surface of the back	Control of melanophore distribution	[290]
	mc1r	Melanocortin receptor 1	CRISPR-Cas9	Pigmentation	The number of melanophores is reduced, the existing cells do not grow even with prolonged exposure to sunlight, the process of melanin formation is disrupted, the fish either acquire a grayish color or become albinos with deformed (shrinked) melanophores	Control of melanin synthesis and melanophore formation	[291]
	tyrp1	Tyrosinase 1	CRISPR/Cas9	Pigmentation	Gray or brown skin color	Control of melatonin synthesis	[292]
**Goldfish**	*tyr*	Tyrosinase	CRISPR/Cas9	Pigmentation	Decreased melatonin synthesis, reduction in body color and decreased expression of other pigmentation-related genes (*tyrp1, mitfa, mitfb, dct, sox10*)	Control of melatonin synthesis	[293]
**Gibel carp**	*cgfoxl2a-B*<br>*cgfoxl2b-A*<br>*cgfoxl2b-B*	Forkhead box protein subfamily FoxL	CRISPR/Cas9	Sex	In polyploid females, knockout of the *cgfoxl2a-B* gene led to underdevelopment of eggs and sex reversal. Knockout of the *cgfoxl2b-A* and *cgfoxl2b-B* genes leads to the absence of germ cells	Genes *cgfoxl2a* and *cgfoxl2b* control folliculogenesis and gonad differentiation<br>The *cgfoxl2b* gene is involved in the regulation of oogenesis	[327]
	cgRunx2b-A<br>*cgRunx2b-B*	runt-related transcription factor 2b	CRISPR/Cas9	Development	With the simultaneous knockout of two genes, there was a reduction in the number of intermuscular	Control of intermuscular bone formation	[244]

	bones in F0. In the F1 gynogenetic generation, 291 of 432 offspring were deprived of intermuscular bones and 80 were partially deprived	
